# Organ-Specific Surveillance and Long-Term Residency Strategies Adapted by Tissue-Resident Memory CD8^+^ T Cells

**DOI:** 10.3389/fimmu.2021.626019

**Published:** 2021-02-15

**Authors:** Jens V. Stein, Nora Ruef, Stefanie Wissmann

**Affiliations:** Department of Oncology, Microbiology and Immunology, University of Fribourg, Fribourg, Switzerland

**Keywords:** tissue-resident T cells, epidermal barrier, salivary gland, chemokine, integrin

## Abstract

Tissue-resident CD8^+^ T cells (CD8^+^ T_RM_) populate lymphoid and non-lymphoid tissues after infections as first line of defense against re-emerging pathogens. To achieve host protection, CD8^+^ T_RM_ have developed surveillance strategies that combine dynamic interrogation of pMHC complexes on local stromal and hematopoietic cells with long-term residency. Factors mediating CD8^+^ T_RM_ residency include CD69, a surface receptor opposing the egress-promoting S1P1, CD49a, a collagen-binding integrin, and CD103, which binds E-cadherin on epithelial cells. Moreover, the topography of the tissues of residency may influence T_RM_ retention and surveillance strategies. Here, we provide a brief summary of these factors to examine how CD8^+^ T_RM_ reconcile constant migratory behavior with their long-term commitment to local microenvironments, with a focus on epithelial barrier organs and exocrine glands with mixed connective—epithelial tissue composition.

## Introduction

During viral infections, Ag-specific naïve CD8^+^ T cells (T_N_) become activated in reactive secondary lymphoid organs (SLOs), and change their gene expression pattern and metabolism to differentiate into proliferating cytotoxic effector T cells (T_EFF_) (1, 2). During the effector phase, T_EFF_ are subdivided into KLRG1^+^ CD127^−^ short-lived effector T cells and KLRG1^−^ CD127^+^ memory precursor effector cells, with a larger potential to generate long-lived memory cells in the latter compartment (3). T_EFF_ killing of infected cells in inflamed tissue requires direct cell-to-cell contact to identify cognate peptide major histocompatibility complexes (pMHC) on target cells, which leads to release of granzymes and perforin for induction of apoptosis (4, 5). Once intracellular infections have been cleared, memory CD8^+^ T cells patrol the body for rapid protective recall responses upon secondary pathogen encounter. Depending on their surface marker expression and trafficking patterns, distinct subsets of memory CD8^+^ T cells are classified (6). Central memory T cells (T_CM_) maintain the ability to recirculate through SLOs through expression of the homing receptors L-selectin (CD62L) and the chemokine receptor CCR7, a characteristic shared with T_N_. Recent work has shown that T_CM_ can also be rapidly recruited to sites of inflammation outside lymphoid tissue (7). Effector memory T cells (T_EM_) lack CD62L and CCR7 expression and are thought to patrol non-lymphoid tissues (NLTs), although their precise functions are still not well-defined (8). Peripheral memory CD8^+^ T cells (T_PM_) have been recently described based on intermediate expression of the chemokine receptor CX3CR1 as predominant subset surveying NLTs (9). Finally, self-renewing, non-recirculating tissue-resident memory T cells (T_RM_) populate barrier organs after clearing of an infection as first line of defense, both in mice and humans (10–17). In contrast to circulating memory T cell subsets, T_RM_ are in a disequilibrium with blood as they are retained for months or years within their tissue of residency. Recent data suggest that tissue-residency vs. circulating memory potential is already imprinted during priming in lymphoid tissue. Migratory dendritic cells (DCs) from skin and gut epithelium present active transforming growth factor (TGF)-β to recirculating CD8^+^ T_N_, which preconditions these cells to form T_RM_ in a skin vaccination model (18). Such conditioning is another example of lymphoid tissue-directed steering of ensuing immune responses, such as reported for differential homing receptor induction in skin-vs. gut-draining lymphoid tissue (19). In line with this observation, a tissue-resident gene expression signature is readily detectable in early circulating T_EFF_ cells prior to entry into NLTs (20). Notably, presence of cognate antigen at infiltrated target sites is not a prerequisite for T_RM_ formation, although it increases their local abundance (21). Finally, in addition to sites of microbial infection, CD8^+^ T cells with a T_RM_ signature are also detectable in tumors and in autoimmune inflammatory conditions, where these cells exert protective and detrimental effects, respectively (17).

Studies following the development of epidermal CD8^+^ T_RM_ have shown that KLRG1^−^ precursor cells enter the dermis during the early effector response and that their entry into the epidermis involves the action of keratinocyte-secreted chemokines that bind to CXCR3 and CCR10 expressed on skin-homing T cells (22, 23). The cytokines IL-15 and TGF-β are involved in the formation and survival of epidermal T_RM_. In particular, TGF-β transactivation by keratinocytes increases expression of the integrin chain CD103, which plays a role in tissue retention of epidermal T_RM_ (see below) (22, 24, 25). T_RM_ are characterized by a core transcriptional program mediated by the transcription factors Hobit and Blimp1, as well as Runx3 and Notch (26–28). As a local adaptation to the lipid-rich skin environment, fatty acid metabolism, and mitochondrial functions regulate epidermal T_RM_ development and survival (29). In addition to epithelial barriers, T_RM_ have been identified in virtually all organs including central nervous system (CNS), exocrine glands, lungs, liver, kidney, bone marrow, reproductive tract, as well as tumors (10, 17, 30–36). Notably, far from being a homogeneous population, T_RM_ display considerable heterogeneity (37–39) and interact with diverse, undefined non-hematopoietic cells during local reactivation (40). Furthermore, a recent report using a Hobit expression/fate reporter mouse line has uncovered that T_RM_ have the capacity to de-differentiate to T_EFF_, which occurs in parallel to Hobit downregulation after TCR activation (41).

**Figure 1:**
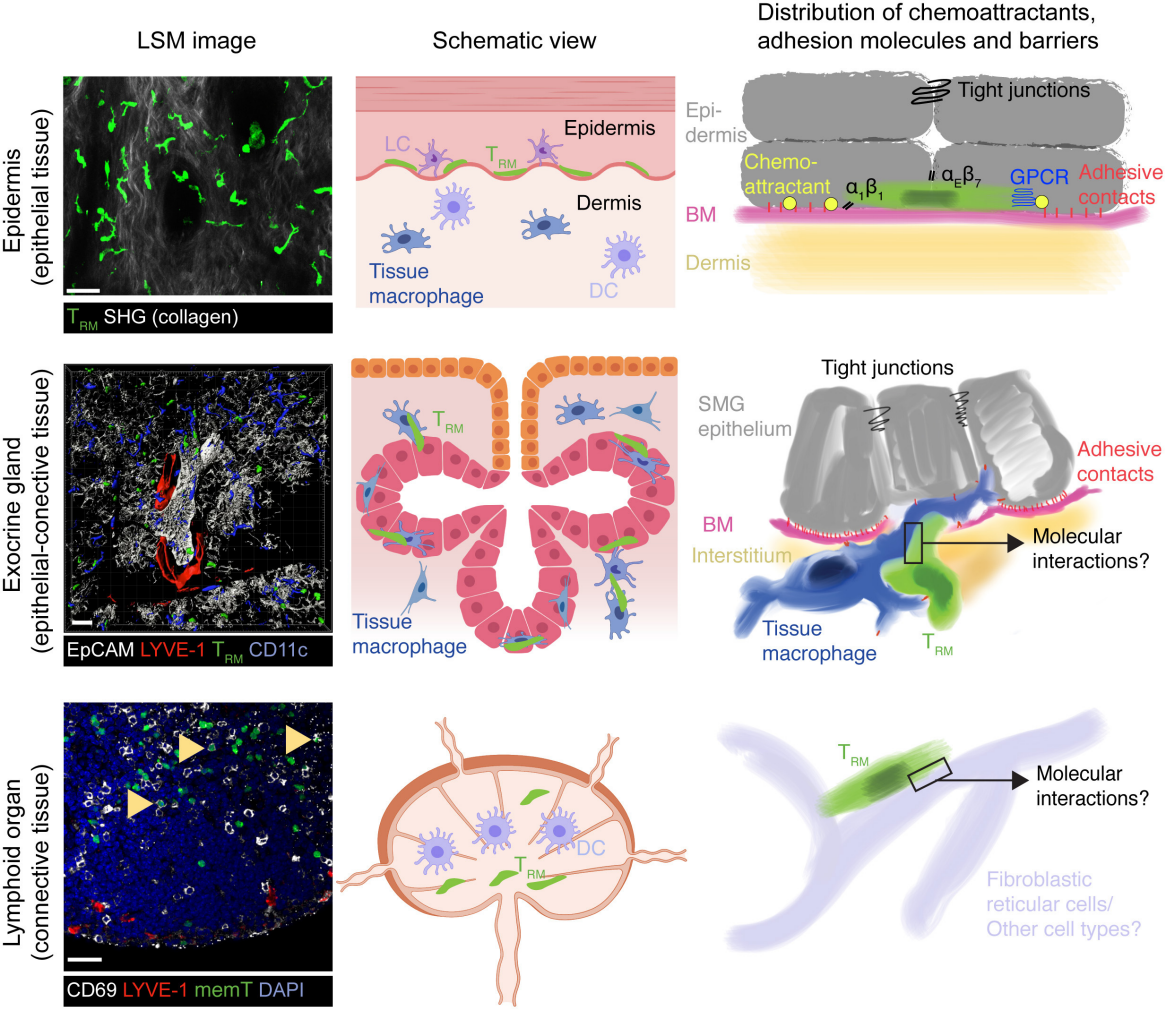
Model of T_RM_ surveillance strategies according to organ topography. In epithelial barrier tissues such as epidermis, T_RM_ mainly locate on top of the basement membrane (BM) separating connective tissue from the epithelium, which themselves are connected by adherens and tight junctions. Both BM and tight junctions serve as physical boundaries to T_RM_ foraging, essentially restricting their motility to a 2D-like surface. Chemoattractants, either constitutively expressed or induced by microbial presence, together with α1 and αE integrins further re-enforce this restricted migration pattern to ensure long-term retention by preventing inadvertent loss of scanning T_RM_ outside the epithelial barrier. In exocrine glands such as the SMG (mixed arborized epithelial—connective tissue), tight junctions between secretory epithelial cells may constitute a similar barrier to prevent loss of T_RM_ into the acini or duct lumen. Yet, the BM separating secretory epithelium from supporting interstitium remains permissive for two-way traffic into and out of epithelial cell layers, which is facilitated in SMG by tissue macrophages. Accordingly, non-inflamed secretory epithelial cells presumably secrete only low levels of chemoattractants that would otherwise retain T_RM_ in this site. This mode of tissue scanning permits rapid accumulation of T_RM_ to sites of secondary pathogen encounters, which would be hampered if T_RM_ were confined exclusively to the epithelial cell layer. While CD69^+^ memory CD8^+^ cells also locate to lymphoid tissue following a viral infection (arrowheads), their function and dynamic interactions with local cells enabling their long-term retention and host protective capacity remain unknown. Similarly, it remains unclear whether SLO T_RM_ retain responsiveness to inflammatory chemokines as their counterparts in epithelial layers and exocrine glands. All confocal images show GFP^+^ OT-I CD8^+^ TCR transgenic T cells at >30 days following systemic or local (skin) virus infections. LSM, laser scanning microscope; SHG, second harmonic generation; LC, Langerhans cells; DC, Dendritic cells; BM, basement membrane, memT, CD8^+^ memory T cells. Scale bar LSM images, 30 μm. Middle panels created with https://biorender.com/.

The localization of T_RM_ to sites of previous pathogen infection poise them to rapidly respond to secondary infections. Accordingly, T_RM_ release cytokines after activation and express high levels of effector molecules such as granzyme B for target cell killing. The protective role for T_RM_ is exemplified by studies in barrier sites of the skin and mucosal surfaces such as the female reproductive tract, where these cells lodge within the epithelium. Antigen re-challenge experiments have shown that T_RM_ act as first-line defense by inducing a tissue-wide alert state, in part via IFN-γ secretion (42–48). These signals relay to innate immune cells for additional cytokine release that results in recruitment of immune cells to the site of pathogen re-emergence, essentially reversing the paradigm that activation of the innate immune system always precedes the adaptive immunity activation. Thus, while T_RM_ also undergo bystander activation through inflammatory cytokines (49, 50), local immune surveillance for cognate pMHC presented on host cells is a key feature of CD8^+^ T_RM_ cells to provide pathogen-specific, long-lasting host protection. To achieve this extraordinary feat, CD8^+^ T_RM_ acquire the ability to infiltrate and physically scan their environment for infected cells within virtually any host organ, while avoiding inadvertent tissue exit via blood or lymphatic vessels or out of an epithelial barrier. Accordingly, CD8^+^ T_RM_ have been found to be patrolling vascular compartments, such as liver sinusoids (51), as well as neuronal and muscle tissue (32, 52). Other anatomical locations surveilled by T_RM_ vary in their content of epithelial and connective tissue: (i) predominantly epithelial (e.g., epidermis and mucosal epithelium), (ii) mixed epithelial—connective (e.g., exocrine and endocrine glands), and (iii) predominantly connective tissue (e.g., lymph nodes and spleen) (Figure 1). Here, we will provide a brief overview on tissue retention and surveillance strategies focusing on data gained in mouse models of skin vs. salivary glands as prototypical epithelial barrier site vs. exocrine gland.

## Multiple Layers of Tissue Retention Cooperate for Long-Term T_RM_ Surveillance of Epithelial Barrier Tissue

Expression of CD69 is the most commonly employed marker to define T_RM_ in all locations, although it is not an exclusive T_RM_ marker and its expression does not necessarily correlate with establishment of long-term resident T_RM_ populations (53, 54). CD69 is a cis-antagonist of the sphingosine-1-phosphate receptor 1 (S1P1) required for egress via lymphatic vessels, which drain interstitial fluid from organs and which contain higher amounts of S1P than tissue (55, 56). T_RM_ also reduce S1P1 production on a transcriptional level, which is prerequisite for establishing long-term residency (57). In epithelial tissues, most T_RM_ express CD103, which is the α_E_ chain of the E-cadherin receptor α_E_β_7_ (6, 58). E-cadherin is expressed by epithelial cells, where it promotes their homotypic adhesion. In line with this, CD103 promotes the long-term persistence of T_RM_ in skin, presumably by retaining these cells within the keratinocyte layer (22). Epidermal CD8^+^ T_RM_ further upregulate the collagen receptor α_1_β_1_, which also contributes to their long-term permanence (59, 60). Finally, T_RM_ increase expression of the negative regulator of chemoattractant receptor signaling, regulator of G-protein-coupled signaling 1 (RGS1) (61, 62). RGS1 and related members of the RGS family activate the GTPase activity of GTP-bound Gα_i_, which leads to a cessation of Gα_i_-coupled receptors signaling (63). RGS-mediated blunted responsiveness to chemoattractants, such as S1P, likely contributes to long-term residency, although experimental evidence is still lacking. Taken together, CD8^+^ T_RM_ have multiple molecular modules at their disposal that in combination reduce the probability to accidentally exit their tissue of residency during homeostatic surveillance. Moreover, the structure of the epithelial microenvironment likely contributes to long-term retention of T_RM_. Epidermal T_RM_ lodge on top of a dense basement membrane (BM) separating underlying connective tissue from the overlying epithelium, and such BM form physical barriers that limit leukocyte dissemination (64). At their apical border, epithelial cells are attached via tight junctions that form a barrier for T cell exit out of the epidermis or into the gut lumen, respectively (65, 66). These factors likely help epithelial T_RM_ to establish long-term tissue-residency as a prerequisite for life-long protection at previously infected sites (Figure 1).

Within their tissue of residency, epidermal T_RM_ physically scan the local cell neighborhood for cognate pMHC. During this process, they display characteristic elongated shapes with numerous dendrites that constantly extend and contract and move in a Gα_i_-dependent manner with speeds of 1–2 μm/min along the bottom keratinocyte layer, resembling motility on a 2D layer (23, 67, 68). Reconstruction of T_RM_ motility in human skin biopsies revealed that these cells occasionally traversed the papillary dermis, and are therefore less strictly confined to the epidermis as observed in mouse skin (69). Both T_RM_ dendricity and motility contribute to efficient scanning of the epidermis (67). Lack of neither the skin-selective chemokine receptors CCR8 or CCR10 (70), nor CXCR3 or CXCR6 affect baseline motility of epidermal T_RM_, although lack of CXCR6 reduces T_RM_ dendricity (23). During secondary viral spread, epidermal CD8^+^ T cells use CXCR3 to follow local chemokine signals and accumulate around infected cells (4, 48). In sum, epidermal T_RM_ maintain responsiveness to inflammatory chemokines despite their Gα_i_-dependent basal motility, suggesting that these chemoattractants override their homeostatic, as yet undefined GPCR input.

Lack of the α_1_β_1_ integrin but not CD103 leads to a loss of the dendrite-shaped T_RM_ morphology (23, 60), suggesting that these cells form transient anchors with their protrusions interacting with extracellular matrix. The precise molecular composition of these transient α_1_β_1_-mediated adhesions remains to be characterized but they likely differ from the more long-lasting anchoring of tissue macrophage protrusions (71). Furthermore, *ex vivo* migration analysis of lung T_RM_ uncovered a role for CD49a in facilitating T_RM_ translocation, whereas CD103 did not promote motility (72). Instead, lack of CD103 leads to an increase in epidermal T_RM_ speeds *in vivo*, suggesting a primary role for this integrin in tissue retention (23). The impact of CD49a on *in vivo* T_RM_ motility parameters has not been determined yet.

Similar to CD49a deficiency, microtubule network depolymerization following nocodazole treatment leads to a loss of the characteristic T_RM_ dendricity (23). This phenomenon is likely due to global release of Rho-activating factor ArhGEF2 otherwise trapped in microtubules (73). Controlled release of ArhGEF2 from depolymerizing microtubules has been recently shown to play an important role in retracting protrusions that are not following the nuclear translocation path during amoeboid cell displacement (74). This pathway serves therefore as a proprioceptive mechanism to control amoeboid cell shape in complex environments such as formed by the tightly packed keratinocyte layer, and is essential to avoid accidental cell rupture. A role for ArhGEF2 in facilitating epidermal T_RM_ motility has thus far not been experimentally addressed. Taken together, continuous retention of epithelial T_RM_ is mediated by multiple integrin receptor interactions and homeostatic GPCR signaling. Long-term T_RM_ colonization may be further facilitated by “layered” architecture of epidermis with a BM separating the underlying connective tissue and the tight junction seal on the apical part of the epithelial layer (Figure 1).

## T_RM_ Lodging and Surveillance of “Non-Barrier” NLTs

In addition to the well-studied epidermis and small intestinal epithelium that are constitutively exposed to microbes, T_RM_ lodge to organs that are less subjected to constant microbial challenge and contain few or no E-cadherin-expressing epithelial layers. These organs include CNS, kidney, submandibular salivary glands (SMG), liver, and bone marrow (10, 16, 75, 76). In contrast to epidermis where CD8^+^ T_RM_ are embedded between non-vascularized epithelial cells, these complex organs contain extensive blood and lymphatic vascular systems, innervation, fibroblasts, tissue-resident macrophages, and innate immune cells, as well as in some cases arborized secretory epithelium. In addition to distinct tissue-specific cellular composition (e.g., kidney tubular cells, hepatocytes, CXCL12-abundant reticular cells of the bone marrow) and receptor-ligand expression patterns, these organs differ in their metabolic activity (e.g., liver) or immunosuppressive environment (e.g., reproductive tract) (77, 78). Furthermore, beyond the biochemical and cellular properties of individual tissues, physical parameters such as topography, substrate stiffness, and confinement influence cell-based immune responses and cross-talk with their environment (79, 80). To date, little is known about how the local microenvironment in these organs affects the phenotype and mechanism of surveillance of T_RM_ during homeostasis and recall responses. While the high expression of CD69, CD49a, and RGS1 on a majority of non-barrier NLT T_RM_ suggests similar roles as in epithelial barrier tissues, CD103 expression is not required for long-term retention of T_RM_ in SMG, in contrast to skin (81, 82). Another key issue is whether memory T cells from distinct anatomical locations employ tissue-specific mechanisms of host surveillance.

**Figure 2:**
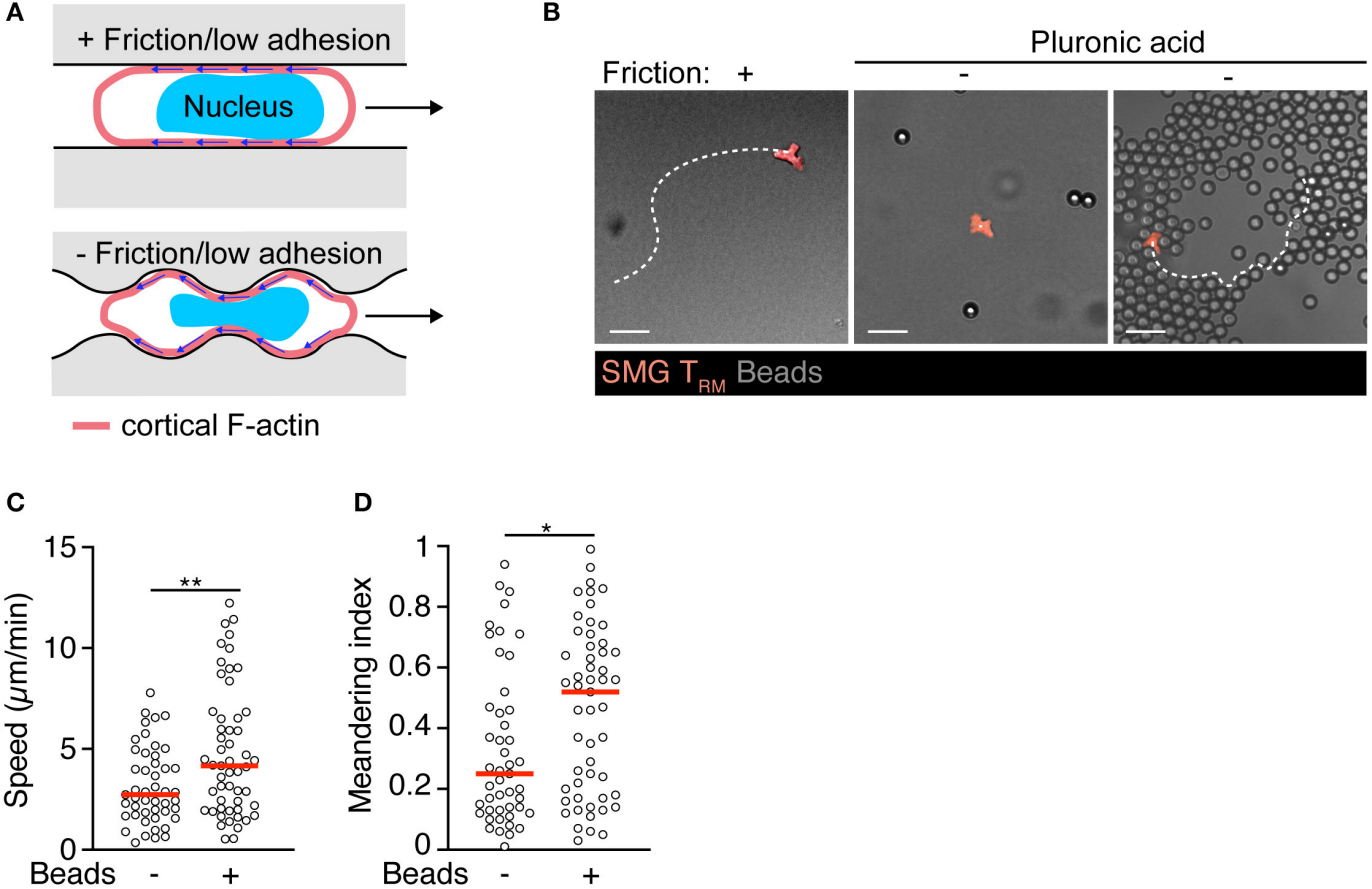
Intrinsic motility of SMG T_RM_ triggered by environmental topography. **(A)** Model for autonomous exocrine gland T_RM_ motility generated by baseline retrograde F-actin flow coupled via non-specific substrate friction or low adhesiveness under physical confinement. In addition, under completely non-adhesive conditions, cell propulsion can be generated through bending of the retrograde cortical actin flow by the environmental topography. Adapted from Reversat et al. (88). (**B)** Exemplary track of isolated SMG T_RM_ in “under agarose” confinement on human serum albumin with and without pluronic acid (PA) passivation to abolish residual friction or lodged between 7 μm-polystyrene bead clusters. Scale bar, 20 μm. **(C)** T_RM_ speeds within or outside of polystyrene bead clusters in presence of PA. **(D)** T_RM_ meandering index within or outside of polystyrene bead clusters in presence of PA. Data were analyzed by unpaired *t*-test **(C)** or Mann–Whitney test **(D)**. *p < 0.05; **p < 0.01.

In a recent study, we have found that T_RM_ lodging in SMG acquire a motility program distinct from T_CM_ and epidermal T_RM_ (83). In contrast to memory T cells isolated from lymphoid tissue or epidermis, *in vivo* observations suggested SMG CD8^+^ T_RM_ were largely refractory to pharmacological inhibition of Gα_i_-protein-coupled receptors or integrin adhesion molecules during homeostatic tissue surveillance, although they retained the ability to respond to inflammatory chemokines and expressed high levels of the CD103, CD49a, CD49d, and CD11a integrins (83). While integrin-independent migration in 3D matrices has become a widely accepted concept in cell biology based on studies with cell lines and DCs (84), several studies demonstrated integrin involvement during immune surveillance of skin T cells (23, 85). As direct evidence for specific adhesion-independent motility, T_RM_ isolated from salivary glands displayed spontaneous motility under 2D confinement in the absence of integrin ligands or chemoattractants. Adhesion-free motility in 2D conditions was reported for large, blebbing carcinoma cells, based on non-specific friction mediated by a large interface between migrating cells and substrates (Figure 2A) (86, 87). Similarly, we observed that non-specific substrate friction is sufficient to trigger intrinsic SMG T_RM_ motility in 2D confinement (83). In turn, T_RM_ isolated from salivary glands did not show displacement on “slippery surfaces,” i.e., in presence of EDTA or when surfaces were passivated with pluronic acid, which reduces friction below a threshold for cell translocation (Figures 2B–D). Notably, these cells regained the capability to translocate in absence of substantial friction when a 3D geometry was created by immotile neighboring objects (Figures 2B–D). This motility mode correlated with continuous changes in cell shapes during migration through microchannels formed by the microenvironment. In this setting, SMG T_RM_ continuously form multiple simultaneous protrusions that probe the environmental geometry, leading to their insertion into permissive gaps and subsequent cell body translocation (83). In the complex 3D exocrine organ architecture, tissue macrophages embedded within the epithelial and connective tissue compartments contributed to generate available extracellular space for protrusion-forming T_RM_ (83).

How do T_RM_ shape changes generate tractive force for cell translocation under these conditions? A recent study has identified adhesion-free cellular locomotion driven by microenvironmental architecture (Figure 2A) (88). Thus, a permissive local topography facilitates cell motility by adapting the cell shape to features of the environment such as crevices and serrated surfaces. At these non-smooth surfaces, rearward cortical F-actin flow generates non-normal forces that results in forward cell motility, rendering cellular translocation autonomous from external influences (Figure 2A). These data provide a model for adhesion-free T_RM_ motility in the absence of friction, and highlight the multiple ways T_RM_ are able to integrate chemical signals (e.g., chemoattractants) and tissue architecture to patrol complex 3D structures such as secretory glands.

What may be the advantages of such a non-canonical migration mode for immune surveillance of mixed connective—epithelial tissues? In contrast to the epidermally restricted migratory behavior of CD8^+^ skin T_RM_ (89), exocrine gland T_RM_ display a bidirectional trafficking pattern into and out of epithelial layers, a process facilitated by tissue macrophages (Figure 1) (83). Such bidirectional trafficking would be perturbed by epithelial chemokine secretion, which could furthermore lead to continuous leukocyte influx and exacerbated inflammation after clearance of infection. Instead, this modus allows T_RM_ to remain responsive to inflammatory chemokines that are locally secreted at sites of pathogen re-emergence. In this context, not being confined to arborized secretory epithelium shortens the pathlength that T_RM_ need to travel in order to accumulate at local sites of inflammation. Furthermore, as ECM proteins and other integrin ligands differ in distinct NLTs (90, 91), integrin-independent motility may endow T_RM_ subsets with flexible topography-driven organ surveillance in non-epithelial barrier sites. A non-proteolytic pathway is beneficial to preserve the integrity of the target tissue, as it does not require constant repair of newly generated discontinuities in the ECM matrix (92). The scanning strategy adopted by homeostatic SMG T_RM_ resembles the migration pattern of T cell blasts in 3D collagen networks, where these cells routinely bypass dense collagen areas, while probing the environment for permissive gaps for cell body translocation (93). In sum, these observations are consistent with a model where certain NLT T_RM_ switch during homeostatic immune surveillance to a self-motile “autopilot” mode supported by tissue macrophage topography, while remaining susceptible to locally produced inflammatory signals for concerted cytotoxic activity. Whether CD8^+^ T_RM_ have adapted a comparable mode for other non-barrier NLTs and whether autonomous motility is shared by other tissue-resident leukocytes, such as CD4^+^ T_RM_, NK or innate lymphoid cells, remains unknown.

## Discussion

Here, we put the general tissue architecture of epidermis and salivary glands as prototype epithelial vs. mixed epithelial—connective tissues into context with published observations on the dynamic surveillance strategies adapted by T_RM_. Reflecting the acknowledged heterogeneity, T_RM_ develop distinct tissue-specific scanning modalities, i.e., chemokine- and integrin-dependent and -independent in epidermis and exocrine glands, respectively, to balance retention and local pMHC interrogation. Independent of their baseline homeostatic migration mode, T_RM_ remain susceptible to inflammatory chemokines produced during pathogen re-encounter, which facilitates their clustering at target sites, perhaps reflecting the low killing rate of cytotoxic CD8^+^ T cells against stromal cell targets (94). Furthermore, certain organs such as epithelial barrier sites might have a higher abundance of promigratory factors in steady state owing to their continuous exposure to microbes. In contrast, non-barrier NLTs may generally express low amounts of chemoattractants in absence of inflammation that demand an adaptation of local immune cells. Recent data suggest that nuclear sensing of confinement may contribute to generate cellular translocation in the absence of external factors (95, 96). Yet, it remains unclear whether or in which NLTs this contributes to T_RM_ surveillance patterns.

A recent observation made by Masopust and colleagues was the presence of *bona fide* CD69^+^ T_RM_ in the red pulp (RP) of spleen and medullary area of LNs (97) (Figure 1), which are at least in part derived from NLT T_RM_ precursors (53). In contrast to CD62L^+^ CCR7^+^ T_CM_ (98), the physiological role of T_RM_ in SLO remains essentially unknown to date. Notably, recent data suggest that in humans a large proportion of memory CD4^+^ and CD8^+^ T cells are CD69^+^ *bona fide* T_RM_, including in LNs and spleen (99). While some of these cells may retain the capacity to recirculate (53), these observations suggest the presence of specific T_RM_ niches with a potential role during re-infection, e.g., via cytokine secretion and/or de-differentiation into T_EFF_ (41). At the same time, the close spatial proximity of spleen T_RM_ to vascular sinuses in the RP (97) raises the question how these cells reconcile dynamic tissue surveillance with long-term retention in a connective tissue with few major tissue barriers such as extensive tight junctions and basement membranes as compared to epithelial barrier sites (Figure 1) (100). Taken together, many incognita remain on the organ-specific T_RM_ cross-talk with the local microenvironment. Combining *in vivo* analysis with high resolution single cell technologies to take into account cell heterogeneity will shed light on these open points.

## Data Availability Statement

The original contributions presented in the study are included in the article/supplementary material, further inquiries can be directed to the corresponding author/s.

## Author Contributions

All authors listed have made a substantial, direct and intellectual contribution to the work, and approved it for publication.

## Conflict of Interest

The authors declare that the research was conducted in the absence of any commercial or financial relationships that could be construed as a potential conflict of interest.
